# Integration of ATAC-seq and RNA-seq analysis identifies host genes related with PRRSV infection

**DOI:** 10.1186/s13567-026-01804-z

**Published:** 2026-07-23

**Authors:** Houchun Liu, Zhong Xu, Hongbo Chen, Xianwen Peng, Liangyu Shi, Cheng Wang, Ao Zhou, Shuqi Mei, Junjing Wu

**Affiliations:** 1 Laboratory of Genetic Breeding, Reproduction and Precision Livestock Farming, School of Animal Science and Nutritional Engineering, Wuhan Polytechnic University, Wuhan, China; 2 Hubei Key Laboratory of Animal Nutrition and Feed Science, Wuhan Polytechnic University, Wuhan, 430023 China; 3 Hubei Key Laboratory of Animal Embryo and Molecular Breeding, Institute of Animal Husbandry and Veterinary, Hubei Provincial Academy of Agricultural Sciences, Wuhan, 430064 China; 4 Xiangxi Tujia and Miao Autonomous Prefecture Animal Husbandry and Aquatic Products Affairs Center, Jishou, 416000 China

**Keywords:** Pig, porcine alveolar macrophages (PAMs), ATAC-seq, RNA-seq, viral infection

## Abstract

The porcine reproductive and respiratory syndrome virus (PRRSV) is a highly contagious pathogen. Viral infections often enhance their replication by modulating the structure and expression of host genes. However, it remains unclear whether PRRSV employs a similar mechanism to achieve self-replication. To address this question, the current study combined assay for transposase accessible chromatin sequencing (ATAC-seq) and ribonucleic acid (RNA) sequencing (RNA-seq) to identify accessible chromatin regions and key host genes associated with PRRSV infection. By comparing the PRRSV-infected group with the control group, we initially detected 8664 differentially accessible chromatin regions and 4037 differentially expressed genes. Motif analysis of these differential chromatin regions revealed several potential cis-regulatory elements containing binding sites for transcription factors. Further integration of ATAC-seq and RNA-seq results identified 1352 overlapping genes between the PRRSV-infected and control groups. A significant positive correlation between differential gene expression and chromatin accessibility signals suggests that chromatin remodeling may drive transcriptional changes during infection. Protein–protein interaction (PPI) network analysis highlighted candidate genes potentially associated with PRRSV infection in hosts, such as *IL1B*, *CCL20*, *CXCL10*, *CSF3*, etc. Given their potential association with the infection mechanism, these genes could serve as candidate targets for the future development of prophylactic vaccines and therapeutic strategies. Additionally, several signaling pathways that may regulate immune and inflammatory responses were significantly enriched in our ATAC-seq and RNA-seq analyses. These findings provide valuable insights into the molecular mechanisms underlying PRRSV infection and pave the way for developing more effective preventive and treatment measures.

## Introduction

Porcine reproductive and respiratory syndrome (PRRS), frequently designated as Blue Ear Disease [1], is an acutely contagious disease that poses a significant threat to the porcine population and is caused by the porcine reproductive and respiratory syndrome Virus (PRRSV). This pathogen predominantly targets the porcine population, eliciting respiratory symptoms across all age groups and inducing reproductive disorders in sows, such as abortions, stillbirths, and reduced postweaning estrus. PRRS is prevalent worldwide, with infected piglets experiencing mortality rates that can soar up to 100% [2]. PRRSV belongs to the order Nidovirales, the family Arteriviridae, and the genus *Arterivirus* [3], which is a single-stranded RNA virus of approximately 50–70 nm in diameter, with an envelope, located in the cytoplasm, and spherical in shape [4]. Notably, PRRSV is characterized by its high genetic variability. It has been further divided into PRRSV-1 (formerly European type I) and PRRSV-2 (formerly North American type II) on the basis of its antigenicity, which share about 60% genetic similarity [5]. The complete genome sequence of PRRSV has been deciphered, with PRRSV-2 strains predominantly circulating in China. Similar to other RNA viruses, PRRSV has been evolving continuously since its emergence, giving rise to numerous variants [6]. Its long incubation period, susceptibility to secondary infections, and high mortality rate pose significant challenges. Moreover, its propensity for mutation and recombination complicates prevention and control efforts [7].

The pathogenesis induced by viral infections is essentially a competitive interplay between the virus and the host. The virus utilizes its encoded proteins to disrupt or hijack the host’s innate immune system, autophagy system, and translation machinery, thereby facilitating viral protein synthesis and genome replication. Conversely, the host employs innate immunity and autophagy mechanisms to inhibit and block key stages of the viral life cycle, thereby thwarting infection [8]. Upon PRRSV infection, the host’s innate immune system acts as the first line of defense. Various immune factors within this system can profoundly influence the course of PRRSV infection and disease progression. After invading the host, PRRSV interacts with innate immune factors through its structural and nonstructural proteins, multiple signaling pathways, and interferon signaling pathways. This interaction enables the virus to evade the host’s immune response, allowing it to complete processes such as adsorption, invasion, phagocytosis, replication, transcription, and translation. Ultimately, this leads to the disruption of the host’s immune system, rapid viral proliferation, and persistent infection [9]. PRRSV can also evade the host’s adaptive immune response through multiple defense mechanisms [10], and antibody-dependent enhancement (ADE) occurs during infection [11]. This phenomenon facilitates viral recognition and entry into target cells, thereby affecting cellular signaling.

In recent years, high-throughput sequencing technologies, particularly RNA-seq, have been widely employed to investigate the molecular mechanisms of PRRSV–host interactions at the transcriptomic level [12]. For example, transcriptome analyses of porcine alveolar macrophages (PAMs) infected with different PRRSV strains have revealed strain-specific pathogenic mechanisms and host immune responses. Studies comparing NADC34-like PRRSV with highly pathogenic PRRSV (HP-PRRSV) demonstrated that different strains induce distinct patterns of inflammatory cytokine and interferon-stimulated gene expression [13]. Similarly, research on HP-PRRSV infection has characterized dynamic gene expression profiles in PAMs, identifying significant dysregulation of genes involved in inflammation, interferon signaling, and antigen presentation pathways [14, 15]. Comparative transcriptome analyses between PRRSV-resistant (e.g., Tongcheng pigs) and PRRSV-susceptible (e.g., Large White pigs) breeds have identified breed-specific differentially expressed genes associated with immune response and apoptosis, providing insights into host genetic determinants of PRRSV susceptibility [16]. Furthermore, integrated analyses of microRNA (miRNA) and messenger RNA (mRNA) transcriptomes have uncovered strain-specific miRNA molecular signatures associated with PRRSV infection, revealing complex post-transcriptional regulatory networks [17]. Beyond PAMs, transcriptomic studies on thymus tissue from PRRSV-infected piglets have revealed that viral infection induces significant thymus injury, with differentially expressed genes enriched in Toll-like receptor, chemokine, and tumor necrosis factor (TNF) signaling pathways [18]. While RNA-seq has been extensively applied to study PRRSV infection, the application of ATAC-seq in this field is still emerging.

Research has unveiled that viral infections induce significant alterations in the chromatin structure of host cells, with certain gene promoter and enhancer regions becoming notably accessible, leading to substantial changes in the host cell gene expression profile [19, 20]. ATAC-seq and RNA-seq analyses of PRRSV-infected cell samples have revealed that many genes in host cells are up- or down-regulated following PRRSV infection of porcine alveolar macrophages (PAMs). These changes suggest that these genes may play critical roles during viral infection. By integrating ATAC-seq results, a set of key genes with significant expression regulation during infection have been identified. These genes are involved in various biological processes, including immune response, metabolic response, inflammatory response, and apoptosis. Functional annotation and pathway analysis have highlighted their important roles during PRRSV infection of PAMs. Some genes are implicated in viral replication and packaging [21, 22], while others are closely associated with the host cell’s antiviral immune response [23–26].

PRRSV exhibits high cell tropism, infecting only monocyte and macrophage lineages in vivo. In vitro, PAMs and peripheral blood monocytes are readily infected, as are certain nonmacrophage cells, such as porcine testicular germ cells and African green monkey kidney cell lines and their derivatives [27]. In addition, a recent study has demonstrated that porcine peritoneal macrophages (PPMs), previously thought to be resistant to PRRSV infection, are in fact susceptible to both PRRSV-1 and PRRSV-2 isolates. This susceptibility is dependent on the expression of *CD163*, the key receptor for PRRSV entry, and is enhanced when cells are cultured prior to infection. These novel findings expand the understanding of PRRSV cellular tropism [28]. In recent years, advancements in high-throughput sequencing technologies have enabled a deeper exploration of the molecular mechanisms underlying viral infection of host cells.

This study has conducted in-depth research into the gene expression regulation of porcine alveolar macrophages (PAMs) infected with the porcine reproductive and respiratory syndrome virus (PRRSV), utilizing the integrated technologies of ATAC-seq (assay for transposase accessible chromatin sequencing) and RNA-seq (transcriptome sequencing). The findings reveal that the infection process entails the regulation of multiple key genes, thereby offering crucial insights and a solid foundation for elucidating the molecular mechanisms underlying PRRSV infection of PAMs and for devising novel antiviral strategies. Moving forward, we will persist in validating the functions of these key genes, with the aim of providing even more valuable information for the prevention and treatment of porcine reproductive and respiratory syndrome (PRRS).

## Materials and methods

### Sample preparation

Healthy 30-day-old *Sus scrofa* domesticus (sourced from Yihezhong Pig Farm, Zhijiang, Hubei Province, China) were humanely euthanized via exsanguination from the femoral artery. A total of 12 pigs were used in this study. For each pig, PAMs were collected as follows: under sterile conditions, the thoracic cavity was carefully opened, and the trachea was meticulously isolated and ligated with a cotton swab. The intact lungs, along with the heart, were then excised. Any blood clots and debris on the lung surface were thoroughly removed using phosphate-buffered saline (PBS). The lungs were placed in an insulated container filled with crushed ice, and 40–50 mL of sterile PBS was instilled into the lungs through the trachea using a pipette. The lungs were gently massaged for 2–3 min to facilitate the distribution of the lavage fluid, which was then aspirated. This lavage procedure was repeated three times until the effluent appeared clear. The collected lavage fluid was filtered through a 70-µm strainer to eliminate other cell types, followed by centrifugation at 1000 rpm for 10 min to pellet the cells. The supernatant was discarded, and the cell pellet was resuspended in 10 mL of PBS (pre-cooled to 4 ℃) containing 2% Penicillin–Streptomycin (Pen Strep). The cells were washed by centrifugation at 1000 rpm for 5 min, discarding the supernatant, and this washing step was repeated twice. The final cell pellet was resuspended in Rosewell Park Memorial Institute (RPMI)-1640 medium supplemented with 10% fetal bovine serum (FBS), and the cell concentration was adjusted to 1.14 × 10^7^ cells/mL. The resulting porcine alveolar macrophages (PAMs) were evenly distributed onto six 6-cm culture dishes and incubated at 37 ℃. After 2 h of cell adherence, the medium was replaced with fresh RPMI-1640 medium containing 10% FBS [29]. Following 24 h of incubation, three dishes from three pigs were randomly designated as the PRRSV infection group (PAM_PRRS), and three dishes from another three pigs were designated as the control group (PAM_CON). The remaining dishes from four pigs were used for other supplementary experiments (e.g., quantitative polymerase chain reaction (qPCR) validation, cell function analysis). The PRRSV infection group (PAM_PRRS) were infected with the PRRSV-HB strain at a multiplicity of infection (MOI) of 0.1, allowed to adsorb for 1 h, and then the medium was replaced with RPMI-1640 medium containing 2% FBS. In the control group (PAM_CON), PAM cells were cultured in RPMI-1640 medium for 1 h before switching to RPMI-1640 medium containing 2% FBS. After 24 h of treatment, cell pellets were collected from both groups. Half of the cell pellet from each dish was directly lysed to construct an RNA-seq library, while the other half was used to construct an ATAC-seq library. Consequently, each of the three RNA-seq biological replicates and each of the three ATAC-seq biological replicates originated from independent pigs, ensuring true biological replication.

### RNA-seq

Raw sequencing reads were processed with Fastp (v0.23.2) for quality control, including removal of adapter sequences and low-quality reads. FastQC (v0.11.9) was used for visualization of the quality control results. The remaining reads were mapped to the pig genome (*Sus scrofa* 11.1) using HISAT2 (v2.2.1) software with default parameters. Differential gene expression analysis was performed between the control group and the infected group using the R package DESeq2. Significantly differentially expressed genes (DEGs) were identified on the basis of the following criteria: |Log_2_(fold change)| > 1 and *p* < 0.05. The experiment included three biological replicates.

### ATAC-seq

A total of six sets of samples were sequenced to construct an ATAC-seq library. For all samples, the raw sequencing reads were processed with cutadapt(v2.5) for quality control and to removallow-quality alignments, cytoplasmic genome alignments, and redundant sequences introduced by polymerase chain reaction (PCR), thereby obtaining high-quality clean reads. The clean reads were then aligned to the reference genome (*Sus scrofa* 11.1) using Bowtie2(v2.3.4.1) software. The aligned sequences were converted from sequence alignment map (SAM) to binary alignment map (BAM) format using Samtools, and these BAM files were used for peak calling. MACS2 (v2.1.1) software was utilized for peak calling to identify open chromatin regions (−*q* 0.05 call peaks-nomodel-shift-100-extsize 200). Regions with a *Q* value less than 0.05 were defined as peaks. ChIPseeker (v1.32.1) was used to evaluate the distribution of peaks across different genomic regions. The Integrated Genomic Viewer (IGV v2.11.7) was employed to visualize all sequencing tracks and bigWig files. Differential peak analysis between groups was performed using the DESeq2 (v1.36.0) package in R, with the criteria of |log_2_(fold change)| > 1 and *p* < 0.05. Homer’s annotatePeak.pl was used to annotate the ATAC-seq peaks. The experiment included three biological replicates.

### Gene functional annotation

Gene Ontology (GO) functional enrichment analysis was conducted to compare genomic backgrounds and identify GO functional terms associated with differentially expressed genes. The results indicated that these differentially expressed genes are closely related to those with important biological functions [30]. Additionally, Kyoto Encyclopedia of Genes and Genomes (KEGG) pathway analysis was performed to provide insights into the metabolic pathways and functions of intracellular gene products [31]. Both GO and KEGG analyses were carried out using the DAVID bioinformatics tool. A *p*-value of 0.05 was set as the significance cutoff for identifying significant GO terms and pathways.

### Integration of protein–protein interaction network and analysis of hub genes

To identify genes with coordinated changes at both the chromatin accessibility and transcriptional levels, we first selected genes that were associated with differential ATAC-seq peaks (at least one significant peak) and were also differentially expressed in RNA-seq. From this overlapping gene set, we applied an additional filter to select genes that exhibited significant changes in both datasets at the gene level. For ATAC-seq, the average chromatin accessibility change across all peaks associated with each gene was calculated, and genes with |log_2_(fold change)| > 1 and *p* < 0.05 were retained. For RNA-seq, genes with |log_2_(fold change)| > 1 and *p* < 0.05 were retained. The resulting gene set was used for subsequent protein–protein interaction (PPI) analysis.

PPI pairs were identified using the STRING database (v12.0) through its PPI retrieval tool, with a minimum required interaction score of 0.4 (medium confidence). The PPI file generated by STRING was then imported into the Cytohuba plugin of Cytoscape 3.10.1 software. The maximum clip centrality (MCC) method was applied to detect the top ten hub genes [32].

### Validate differentially expressed genes in transcriptome data on the basis of qRT PCR results

To validate the relative expression patterns obtained from RNA-seq, we performed quantitative real-time PCR (qRT-PCR) on samples from three infected groups and three control groups. Total RNA was extracted using RZ lysis buffer (Tiangen Biotech, Beijing, China). Subsequently, reverse transcription was carried out using a reverse transcription kit (Vazyme Biotech, Nanjing, China). The resulting complementary DNA (cDNA) was quantified using a 2X Taq Pro Universal SYBR qPCR Master Mix (Vazyme Biotech, Nanjing, China) on a real-time fluorescence quantitative PCR instrument (Thermo Fisher, QuantStudio™ 1 Plus, Waltham, MA, USA). The amplification protocol consisted of 40 cycles, with each cycle comprising the following steps: 95 ℃ for 30 s, followed by 95 ℃ for 10 s, 60 ℃ for 15 s, 60 ℃ for 60 s, and a final step at 95 ℃ for 15 s. The primers used in this study are listed in Table 1. The expression levels of the target genes were normalized to the housekeeping gene *GAPDH*. The experiment included three biological replicates.

**Table 1:** **Primers used to verify the quality of samples**

Name	Primer sequence (5′ to 3′)
*METTL1*-F	CAACCCCATGGCAGACCATA
*METTL1*-R	CCTGTTAGTGGCACTGTCAC
*CSF3*-F	AAGTGCTTAGAGCAAGTGAGGAAA
*CSF3*-R	GCTGCCTGAACCAACTGCAT
*IDO2*-F	TCCAGCAAAATCAGGGGGAC
*IDO2*-R	AGGAATGGATGCCCCTCTTG
*IDO1*-F	GAGGAGCTGCCTCACCCTTA
*IDO1*-R	GAAGTTAGCAACGCTCAGCAT
*IRAK3*-F	AGGATTTCCGCGGTTGTGTA
*IRAK3*-R	CTTGCGGAGCGACTTTCAAG
*BTN1A1*-F	CTCCTGGAAGAGCTCAAATGGA
*BTN1A1*-R	AGGACTGATTGGGCAATCGG
*IFITM3*-F	CTGGTCCCTGTTCAACACCC
*IFITM3*-R	ATCGTTTGCACCACTGGCTC
*MAP2K6*-F	TAAACAGCCAGGAGCAGAAAA
*MAP2K6*-R	CCTTCCAACGTCCTGATCAA
*TIFAB*-F	ACACATCCCCAAACGGTAGC
*TIFAB*-R	GAAGTAAGGCTCCTGTCCATGG
*DPRX*-F	AAAAGTGCCAGAGCCATCCA
*DPRX*-R	GCAGGTTTGGTTTAAGAACCG
*SELENOP*-F	GGAAGACCTGCGAGTAAAAC
*SELENOP*-R	CATCTTGGTTTGCCTTATTCC
*GAPDH*-F	TCGGAGTGAACGGATTTG
*GAPDH*-R	CCTGGAAGATGGTGATGG

### Validation of *CSF3* and *METTL1* genes by small interfering RNA (siRNA) knockdown and viral challenge

To functionally validate the roles of *CSF3* and *METTL1* during PRRSV infection, siRNA-mediated knockdown experiments followed by viral challenge were performed in both MARC145 cells and primary porcine alveolar macrophages (PAMs).

### siRNA design and selection

Multiple siRNA sequences targeting porcine *CSF3* and *METTL1* were designed and synthesized by GenePharma (Suzhou, China). The siRNA sequences used in this study are listed below:

For *CSF3*:

siCSF3-252-s: 5′-GCUUAGAGCAAGUGAGGAATT-3′

siCSF3-523-s: 5′-GCUGGAUGUCACCGACUUATT-3′

For *METTL1*:

siMETTL1-106-s: 5′-GCCUCAGAAGCGCUACUAUTT-3′

siMETTL1-203-s: 5′-GAGCUAUAUCCAGAGUUCUTT-3′

siMETTL1-515-s: 5′-GGCCAGCUGACAAAGAUGUTT-3′

Preliminary screening for knockdown efficiency was conducted in both cell types. The most effective siRNA for each gene was selected for subsequent functional assays. Notably, the optimal siRNA sequences differed between cell lines: in MARC145 cells, the validated effective sequences were siCSF3-252 and siMETTL1-203. In primary PAMs, the validated effective sequences were siCSF3-252 and siMETTL1-515.

The relevance of using porcine-derived siRNAs in the African green monkey-derived MARC145 cells was supported by cross-species homology analysis. Sequence alignment revealed that the coding regions of *CSF3* and *METTL1* share 85.27% and 91.15% identity between *Sus scrofa* (pig) and *Chlorocebus sabaeus* (African green monkey), respectively.

### Viral challenge experiment

For both MARC145 cells and PAMs, three experimental groups were established: negative control siRNA (NC) + PRRSV, siCSF3 + PRRSV, and siMETTL1 + PRRSV. Cells were transfected with the respective validated siRNAs (30 pmol per transfection) using the Seven HighTrans™ transfection reagent (Seven, Beijing, China)according to the manufacturer’s instructions.

After 24 h of incubation at 37 ℃ with 5% CO_2_, the cells were inoculated with the PRRSV-HB strain at a multiplicity of infection (MOI) of 0.1 and further cultured for 36 h under the same conditions.

Post-infection, cells were harvested for downstream analysis. Total RNA was extracted using RZ lysis buffer and the TianGen Total RNA Extraction Kit (Tiangen Biotech, Beijing, China). The viral load was quantified via qRT-PCR targeting the PRRSV *ORF5* and *ORF7* genes. The primers used were:

PRRSV ORF5-F: 5′-ATGCCAAATACAACGG-3′

PRRSV ORF5-R: 5′-TGCTGAGGGTGATGCTGT-3′

PRRSV ORF7-F: 5′-GTTTGTGCTTGCTAGGCCG-3′

PRRSV ORF7-R: 5′-CTGCCACCCAACACGAGG-3′

For protein analysis, total protein was extracted from cell pellets. Protein concentration was determined using a BCA protein assay kit (Vazyme, Nanjing, China). Proteins were separated by sodium dodecyl sulfate-polyacrylamide gel electrophoresis (SDS-PAGE), transferred to polyvinylidene fluoride (PVDF) membranes, and probed with primary antibodies against beta-Actin (Abways, Minhang District, Shanghai China) and PRRSV GP5 protein (Ke’an Biotechnology, Wuhan, China). Protein signals were visualized using a SuperPico ECL chemiluminescence substrate (Vazyme) and captured with a chemiluminescence imaging system. All experiments were performed with at least three biological replicates to ensure reproducibility.

### Graphical workflow

**Figure 1:**
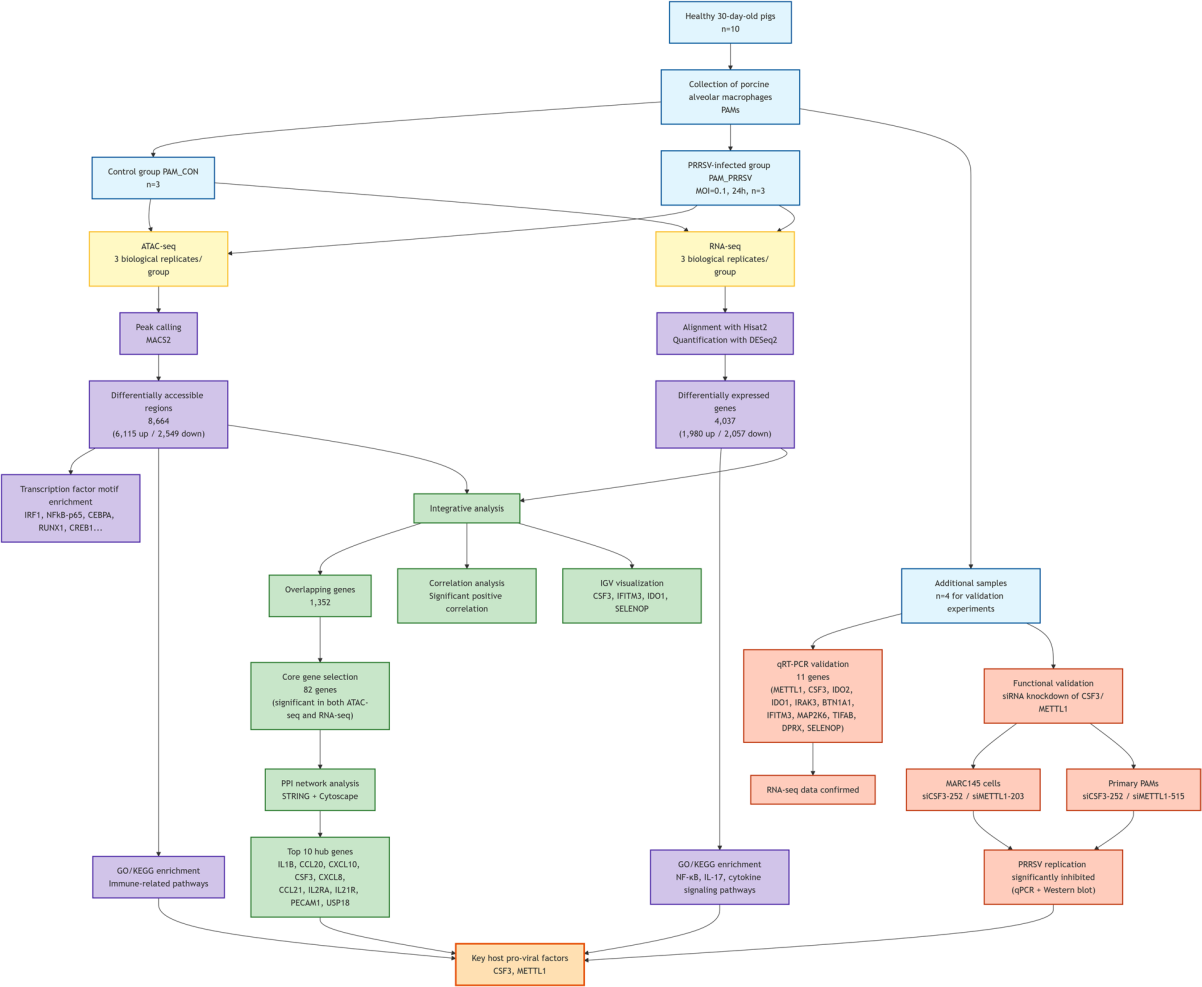
**Graphical workflow of the study design and analysis.**

An overview of the study design and analytical pipeline is presented in Figure 1. Schematic overview of the experimental pipeline used to identify host proviral factors involved in PRRSV infection. Porcine alveolar macrophages (PAMs) were collected from healthy 30-day-old pigs (*n* = 10) and divided into control (PAM_CON, *n* = 3) and PRRSV-infected (PAM_PRRSV, MOI = 0.1, 24 h, *n* = 3) groups, then subjected to ATAC-seq and RNA-seq with three biological replicates per group. ATAC-seq data were processed using MACS2 for peak calling, identifying 8664 differentially accessible regions (6115 up/2549 down), with transcription factor motif enrichment analysis revealing IRF1, NFkB-p65, CEBPA, RUNX1, CREB1, etc. RNA-seq data were aligned with HISAT2 and quantified with DESeq2, yielding 4037 differentially expressed genes (1980 up/2057 down). Integrative analysis identified 1352 overlapping genes, from which 82 core genes were selected. PPI network analysis (STRING + Cytoscape) identified top ten hub genes (e.g., IL1B, CCL20, CXCL10, CSF3, CXCL8), and key host proviral factors CSF3 and METTL1 were selected for further validation. qRT-PCR validation of 11 genes was performed using additional samples (*n* = 4), and IGV visualization confirmed differential expression of CSF3, IFITM3, IDO1, and SELENOP. Finally, knockdown of CSF3 and METTL1 using specific siRNAs (siCSF3-252, siMETTL1-203 in MARC145 cells; siCSF3-252, siMETTL1-515 in primary PAMs) significantly inhibited PRRSV replication, as confirmed by qPCR and western blot.

## Results

### RNA-seq data

**Figure 2:**
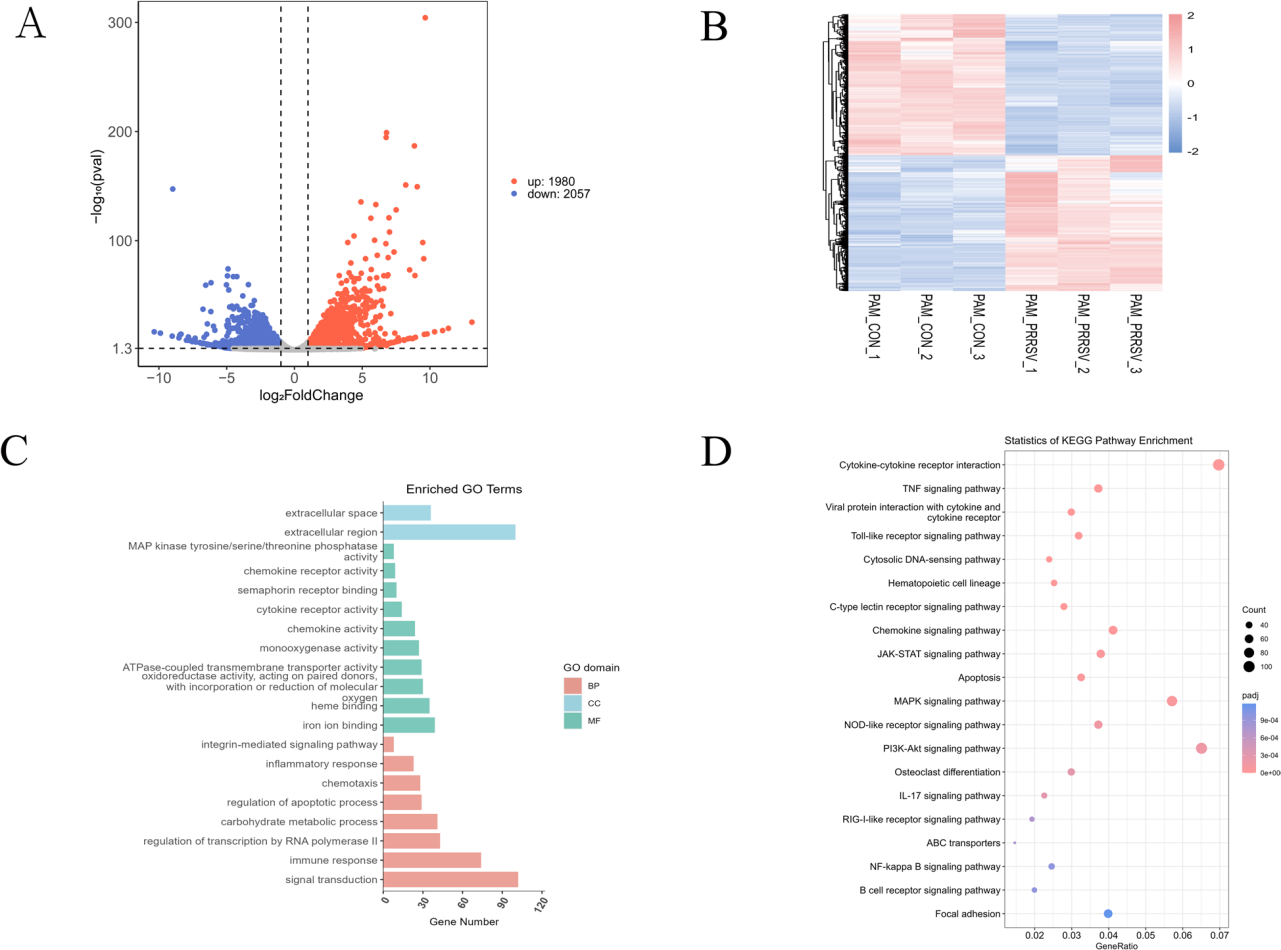
**Analyses of RNA-seq.** **A** scatter plot of the transcriptome data [*p* < 0.05, |log_2_(fold change)| > 1]. **B** heatmap of the differentially expressed genes. **C** GO enrichment analysis of differentially expressed genes. **D** bubble chart of Kyoto Encyclopedia of Genes and Genomes (KEGG) pathway enrichment analysis of differentially expressed genes.

To investigate the transcriptomic alterations in porcine alveolar macrophages (PAMs) following PRRSV infection, we employed RNA-seq technology to analyze the changes after 24 h of infection. A total of six RNA-seq libraries were constructed, and the sequencing results showed that 90.8–94.2% of the clean reads aligned with the pig reference genome (*Sus scrofa*; Table 2). Utilizing the criteria of |log_2_(fold change)| > 1 and *p* < 0.05, we identified a total of 4037 differentially expressed genes (DEGs), comprising 1980 upregulated and 2057 downregulated genes (Figure 2A). A heatmap of these DEGs (Figure 2B) revealed consistent expression patterns within each group and significant intergroup differences. To elucidate the potential functions of these DEGs in PRRSV infection, we conducted Gene Ontology (GO) enrichment analysis and Kyoto Encyclopedia of Genes and Genomes (KEGG) pathway enrichment analysis. The GO analysis indicated that the biological processes were primarily enriched in signal transduction, immune response, and regulation of transcription by RNA polymerase II (Figure 2C). The molecular functions were mainly enriched in iron ion binding, heme binding, and oxidoreductase activity, particularly those acting on paired donors with oxygen incorporation or reduction of molecular oxygen. The KEGG analysis further revealed that the DEGs were significantly enriched in pathways related to immune response, such as the NF-κB signaling pathway [33], IL-17 signaling pathway [34], and cytoplasmic DNA sensing pathway [35] (Figure 2D). These findings suggest that the identified DEGs may be intricately involved in the immune response elicited during PRRSV infection of the host.

**Table 2:** **Summary of the RNA-seq data**

Sample	Raw reads	Raw bases	Clean reads	Clean bases	Q20 (%)	Q30 (%)	Unique mapped reads
PAM_CON_1	44,799,376	6,719,906,400	44,738,524	6,661,741,352	98.36	95.79	42,160,189 (94.24%)
PAM_CON_2	51,715,566	7,757,334,900	51,642,888	7,586,364,126	98.62	96.46	48,601,505 (94.11%)
PAM_CON_3	54,885,584	8,232,837,600	54,809,036	8,064,522,874	98.49	96.14	50,808,300 (92.70%)
PAM_PRRSV_1	52,203,626	7,830,543,900	52,128,534	7,740,671,308	98.23	95.47	48,020,388 (92.12%)
PAM_PRRSV_2	48,950,756	7,342,613,400	48,869,898	7,191,902,982	98.52	96.15	44,392,755 (90.84%)
PAM_PRRSV_3	48,324,850	7,248,727,500	48,248,392	7,180,139,780	98.36	95.76	44,463,161 (92.15%)

### ATAC-seq quality control for PAM cell samples

To investigate the genome-wide alterations in chromatin accessibility in porcine alveolar macrophages (PAMs) upon PRRSV infection, we performed ATAC-seq on both infected and control groups. A total of six high-quality sequencing libraries were constructed. The sequencing results demonstrated high alignment rates, with 85.5–90.9% of the clean reads successfully mapped to the pig reference genome (*Sus scrofa* 11.1), confirming the efficiency of the library preparation and sequencing (Table 3).

**Table 3:** **Summary of the ATAC-seq data**

Sample ID	Raw reads	Raw bases (G)	Raw Q20 (%)	Raw Q30 (%)	Clean reads	Clean bases (G)	Clean Q20 (%)	Clean Q30 (%)
PAM_CON_1	267,571,546	40.14	93.11	84.71	267,555,682	34.67	93.76	85.57
PAM_CON_2	128,090,270	19.21	95.29	89.47	128,067,814	14.02	96.22	90.97
PAM_CON_3	138,115,520	20.72	95.02	88.8	138,104,340	15.57	95.87	90.15
PAM_PRRSV_1	127,260,134	19.09	95.53	89.83	127,252,758	14	96.19	90.94
PAM_PRRSV_2	140,980,826	21.15	94.86	88.92	140,971,902	15.61	95.91	90.61
PAM_PRRSV_3	143,597,664	21.54	95.23	89.28	143,590,302	16.19	96.04	90.69
Total	945,615,960	141.85			945,542,798	110.06		

**Figure 3:**
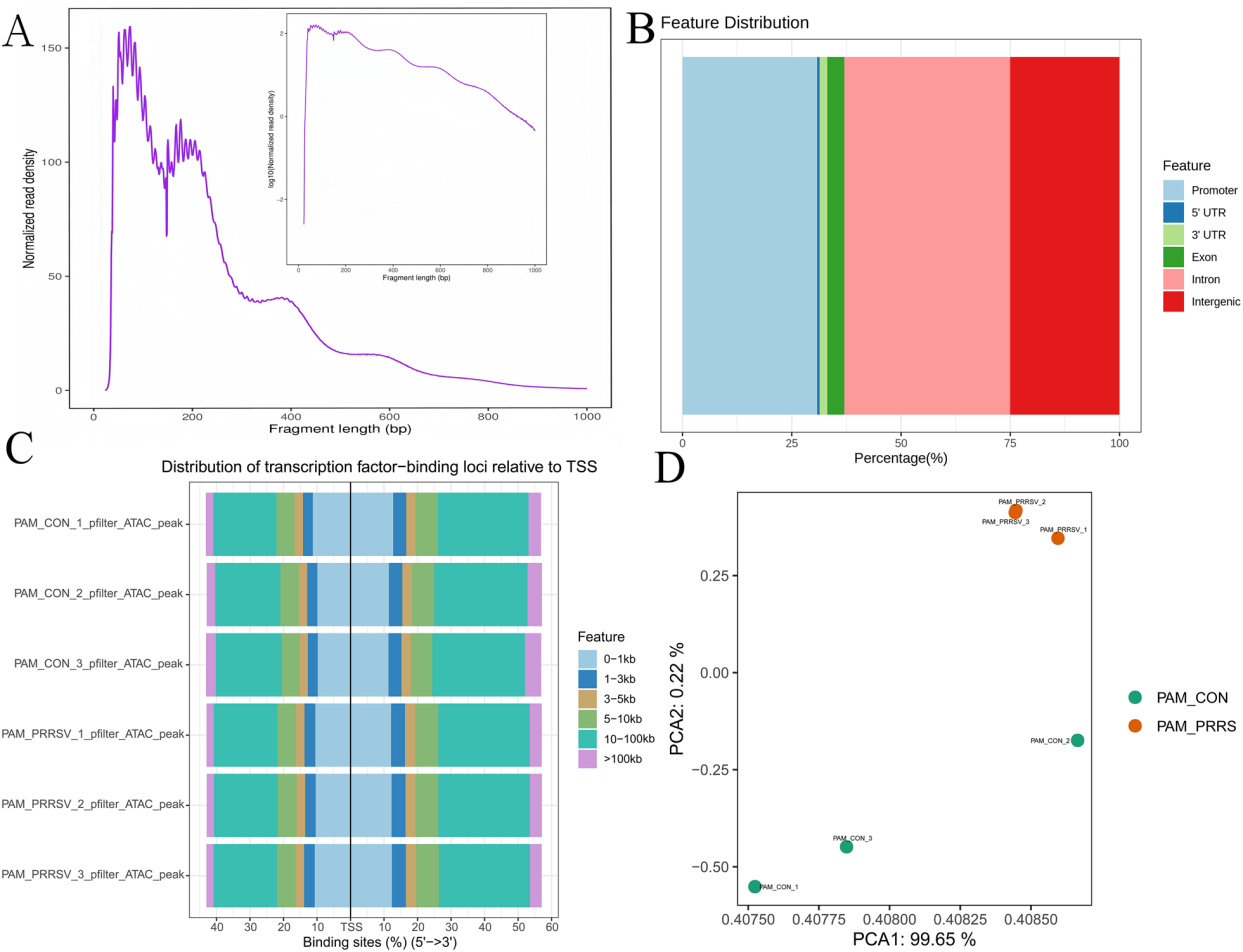
**ATAC-seq quality control and analyses of the peaks.** **A** fragment length distribution map. **B**, **C** mapped reads distributions across gene bodies and peaks. **D** PCA analysis chart based on whole genome alignment.

Rigorous quality control was conducted to ensure the reliability of the data for subsequent analysis. The fragment length distribution of all libraries exhibited a pronounced periodicity, with a dominant peak below 100 bp, corresponding to nucleosome-free regions, and subsequent smaller peaks at approximately 200 bp and 400 bp, representing mononucleosomal and dinucleosomal fragments, respectively (Figure 3A). This characteristic pattern is indicative of high-quality ATAC-seq data with effective Tn5 transposition. Furthermore, the distribution of mapped reads across gene bodies and peak regions was uniform and exhibited the expected patterns, confirming minimal technical bias (Figure 3B, C). Principal component analysis (PCA) of the genome-wide chromatin accessibility profiles revealed clear segregation between the control (PAM_CON) and PRRSV-infected (PAM_PRRSV) groups, with biological replicates within each group clustering tightly together (Figure 3D). This underscores the high reproducibility of the experiments and the pronounced impact of PRRSV infection on the chromatin landscape of PAM cells.

These comprehensive quality control metrics collectively affirm the high quality and reliability of our ATAC-seq datasets, providing a solid foundation for the subsequent identification of differentially accessible chromatin regions.

### Genome-wide identification of accessible chromatin regions

**Figure 4:**
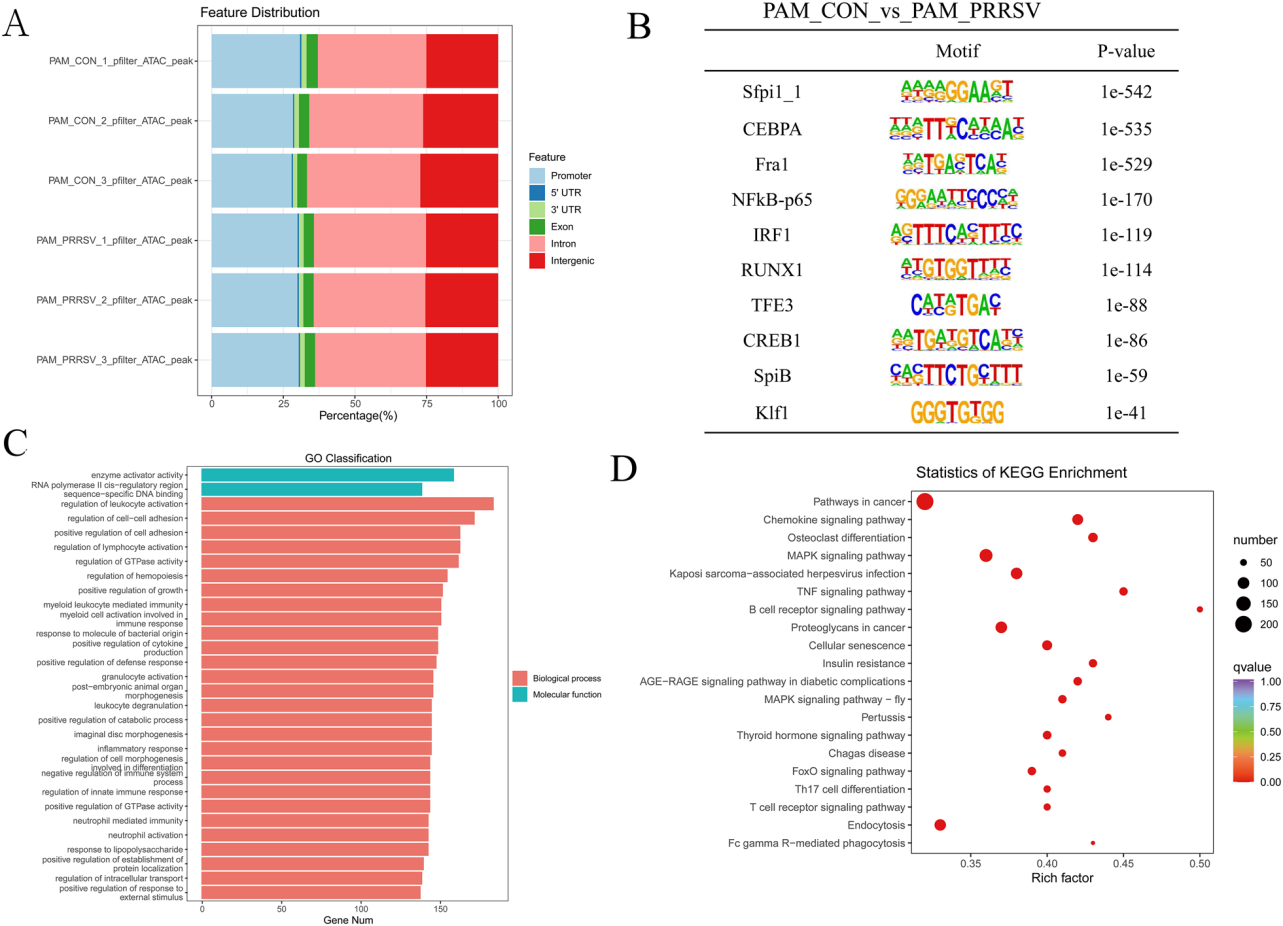
**Distribution of peaks and analysis of differential peaks. A** genomic distribution of the peaks in each sample. Genomic functional regions include promoter, intergenic, exon, intron, 5’UTR, and 3′UTR. **B** enriched transcription factor binding motifs by peaks between the PAM_CON and PAM_PRRSV by ATAC-seq. **C** GO enrichment analysis of genes corresponding to differential peaks. **D** KEGG pathway enrichment analysis of genes corresponding to differential peaks.

We identified 84 640 and 96 113 comparable chromatin peaks in the control group and the infection group, respectively. The genomic distribution of these open chromatin peaks was annotated using reference annotation files. As anticipated, the majority of peaks were mapped to promoters, introns, and intergenic regions (Figure 4A). To pinpoint the open chromatin sites associated with PRRSV infection, we compared the differences in discriminant peaks between the control and infected groups using ATAC-seq. Relative to the control group, the infected group exhibited 6115 upregulated peaks (indicating more accessible chromatin) and 2549 downregulated peaks (indicating less accessible chromatin). The top ten transcription factor binding motifs significantly enriched in PAM_CON compared with PAM_PRRSV include Sfpi1_1, CEBPA, Fra1, NFkB-p65, IRF1, RUNX1, TFE3, CREB1, SpiB, and Klf1 (Figure 4B). For each differentially regulated peak, we identified 2549 downregulated genes and 6115 upregulated genes in the control group compared with the infection group. GO and KEGG pathway enrichment analyses were performed on these genes. All GO terms were categorized into three main types: biological processes (BP), molecular functions (MF), and cellular components (CC) [36]. The top 20 significantly enriched GO terms for the upregulated genes are shown in Figure 4C. The biological processes of GO enrichment primarily focused on immune responses, such as leukocyte activation, regulation of lymphocyte activation, and regulation of cell adhesion. The molecular functions mainly centered on enzyme activator activity and RNA polymerase II cis-regulatory region sequence-specific DNA binding. KEGG enrichment analysis revealed 20 pathways, with smaller *q*-values indicating more significant enrichment. The significantly expressed genes were predominantly concentrated in pathways such as endocytosis, mitogen-activated protein kinase (MAPK) signaling pathway, and Kaposi’s sarcoma-associated herpesvirus infection (Figure 4D).

### Joint analysis of ATAC seq results and RNA seq results

**Figure 5:**
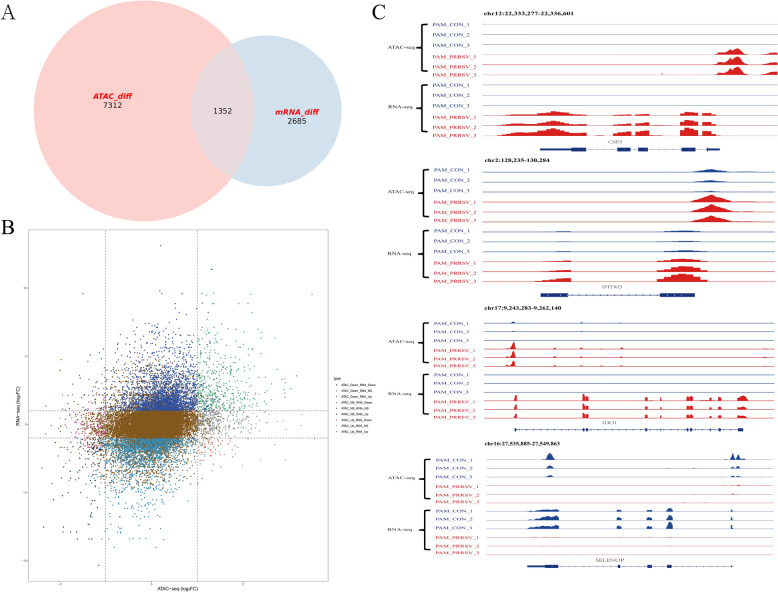
**Integration analysis of ATAC-seq and RNA-seq results.** **A** overlap of differentially expressed genes identified by ATAC-seq and RNA-seq. **B** correlation of significantly differentially accessible gene (ATAC-seq) and gene expression (RNA-seq). **C** IGV snapshot for ATAC-seq and RNA-seq signal for the *CSF3*, *IFITM3*, *IDO1*, and *SELENOP* gene.

To explore the potential correlation between alterations in open chromatin regions and changes in gene expression, we performed an integrated analysis of ATAC-seq and RNA-seq datasets. As shown in Figure 5A, we identified a total of 1352 overlapping genes. Specifically, we examined the correlation between the expression levels of 4037 differentially expressed genes and the accessibility of 8664 differentially accessible chromatin peaks. As illustrated in Figure 5B, a significant positive correlation was observed between changes in chromatin accessibility and changes in gene expression levels. For instance, as depicted in Figure 5C, the chromatin accessibility and transcription levels of *CSF3*, *IFITM3*, and *IDO1* were higher in PAM_PRRSV compared with PAM_CON, whereas the chromatin accessibility and transcription levels of SELENOP were lower in PAM_PRRSV than in PAM_CON.

### Protein interaction network analysis

**Figure 6:**
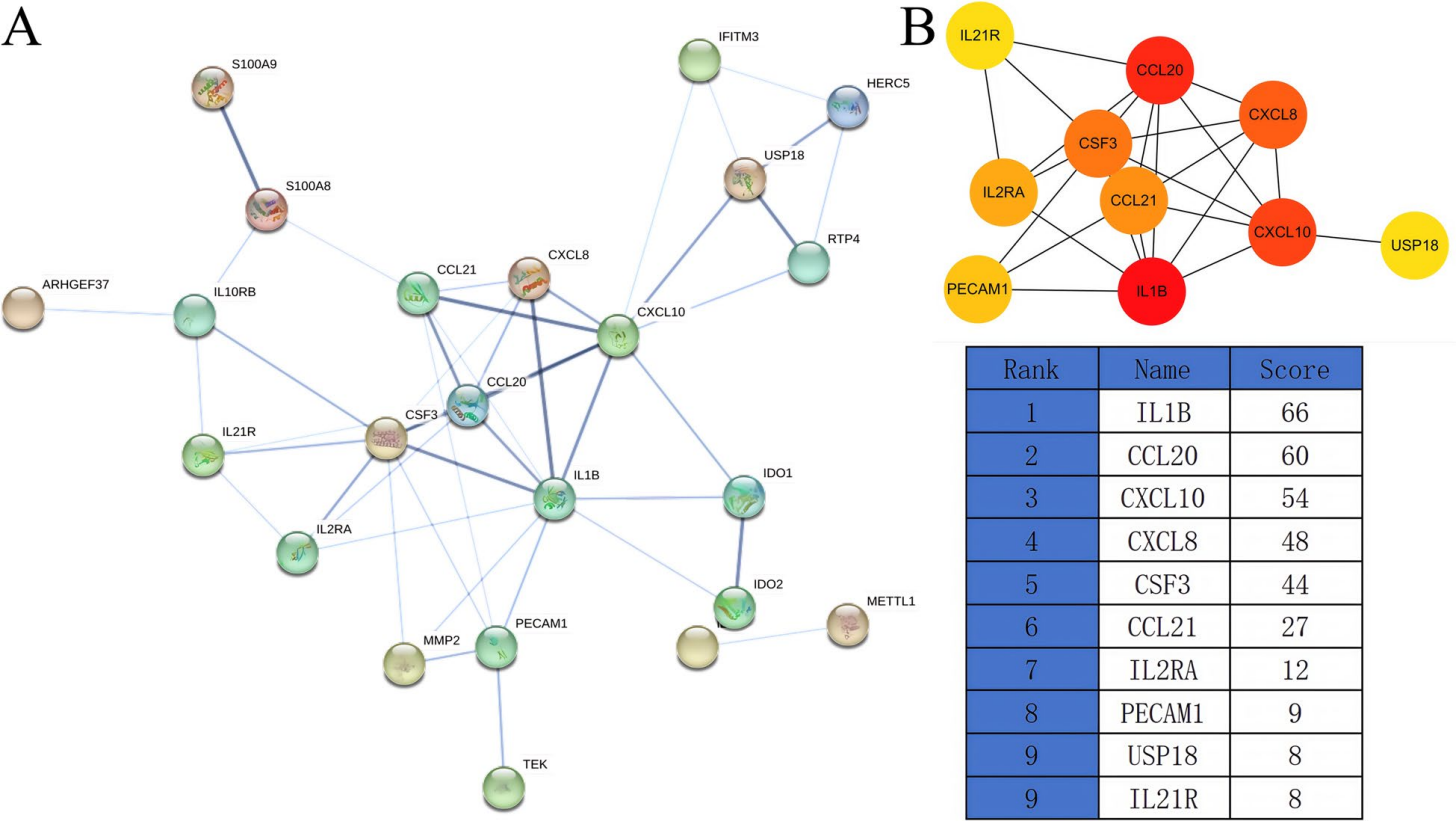
**PPI network of the overlapping genes.** **A** PPI analysis of 82 overlapping genes from PAM_PRRSV versus PAM_CON comparison according to the STRING database. **B** PPI network of the top ten hub genes visualized using Cytoscape.

The protein–protein interaction (PPI) network is a pivotal analytical framework employed to delineate the complex interplay among genes or proteins. This includes both physical associations and regulatory interactions, thereby shedding light on the fundamental molecular regulatory mechanisms that govern biological functions [37]. Within the scope of the present investigation, we leveraged the STRING database (version 12.0) to scrutinize protein–protein interactions, utilizing the R package STRINGdb (version 2.8.4) for our analysis. Our approach involved the integration and analysis of both ATAC-seq and RNA-seq datasets, yielding the identification of 1352 genes that were expressed across both sequencing platforms. We further refined our analysis by excluding genes that lacked significant expression alterations in either of the sequencing platforms. This stringent filtration process led to the identification of 82 genes exhibiting concurrent significant expression changes in both ATAC-seq and RNA-seq, which were subsequently classified as core genes upon their importation into STRING, as depicted in Figure 6A. Subsequently, the extracted data were imported into Cytoscape for network construction, yielding a network architecture that encompassed 23 nodes and 92 edges. Through this network, we pinpointed the top ten nodes with the most robust connectivity within the PPI network, designating them as hub genes. These genes included *IL1B*, *CCL20*, *CXCL10*, *CSF3*, *CXCL8*, *CCL21*, *IL2RA*, *IL21R*, *PECAM1*, and *USP18*, as illustrated in Figure 6B. The identification of these hub genes is instrumental for elucidating the key molecular players in the biological processes under study.

### Validation results via qRT-PCR

**Figure 7:**
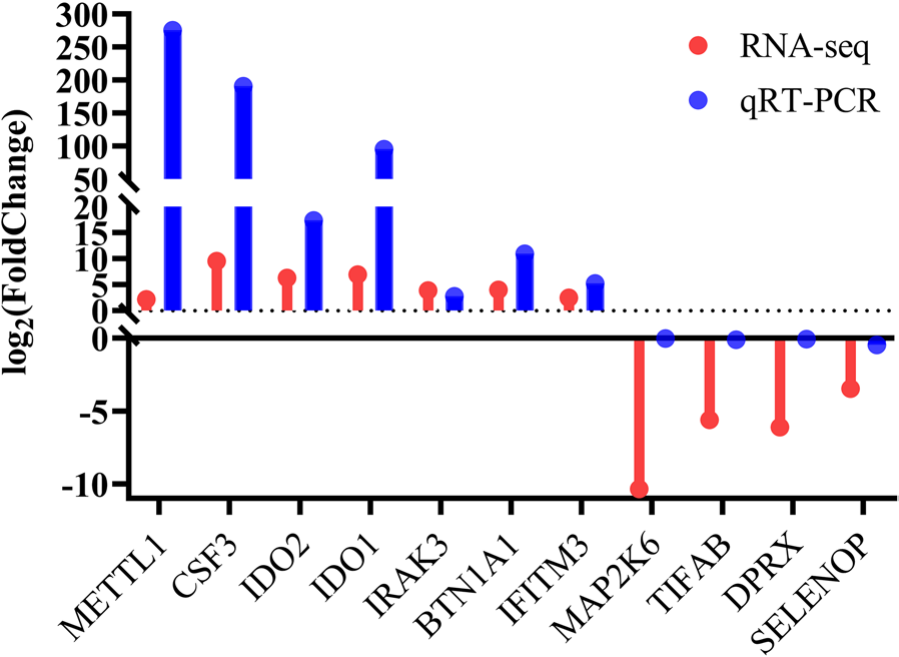
**Verification of RNA-seq data by qRT-PCR.** Histogram of RNA-seq and qRT-PCR expression levels. X-axis represents 11 selected genes, and Y-axis represents the expression levels of genes from RNA-seq and qRT-PCR.

To authenticate the reliability of the RNA sequencing data, a subset of 11 genes—namely *METTL1*, *CSF3*, *IDO2*, *IDO1*, *IRAK3*, *BTN1A1*, *IFITM3*, *MAP2K6*, *TIFAB*, *DPRX*, and *SELENOP*—was randomly selected from the top 20 differentially expressed genes (DEGs) for validation using quantitative reverse transcription polymerase chain reaction (qRT-PCR). The qRT-PCR results substantiated the congruence between the expression profiles determined by RNA sequencing and those obtained through qRT-PCR analysis, thereby validating the accuracy of the RNA-seq data (Figure 7).

### Validation of* CSF3 *and *METTL1* in MARC145 cells

Based on the validation of RNA-seq accuracy, we selected *CSF3* and *METTL1* for further functional investigation during PRRSV infection. We employed siRNA-mediated knockdown in the PRRSV-permissive MARC145 cell line, using the validated porcine-derived siRNA sequences siCSF3-252 and siMETTL1-203, the applicability of which was supported by high cross-species coding sequence homology (85.27% for *CSF3* and 91.15% for *METTL1*).

**Figure 8:**
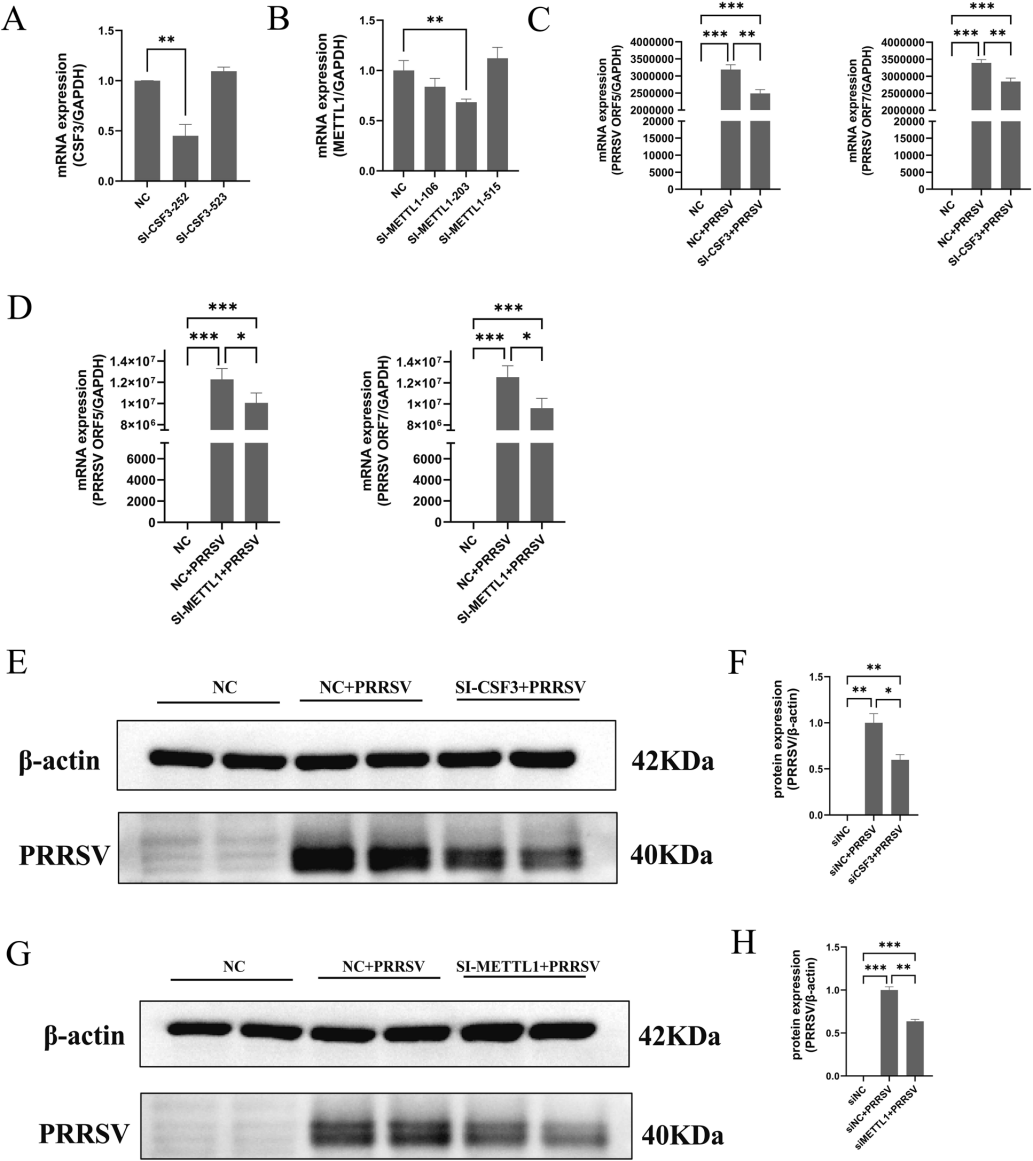
**Knockdown of CSF3 or METTL1 inhibits PRRSV replication in MARC145 cells.** **A**, **B** knockdown efficiency of **A** SI-CSF3 and **B** siMETTL1 in MARC145 cells, as determined by qRT-PCR. **C**, **D** PRRSV mRNA expression levels in MARC145 cells after transfection with **C** siCSF3 or **D** siMETTL1 and infection with PRRSV (MOI = 0.1) for 36 h. **E**–**H** Western blot analysis (**E**, **G**) and corresponding grayscale quantification (**F**, **H**) of PRRSV GP5 protein levels in MARC145 cells after knockdown of **E**, **F** *CSF3* or **G**, **H** *METTL1*. Data are presented as mean ± SD from three independent experiments. *p < 0.05, **p < 0.01, ***p < 0.001 (by Student’s *t*-test). The siRNA sequences used were siCSF3-252 and siMETTL1-203. The apparent molecular weight of the GP5 protein (25–37 kDa) observed here is characteristic of the PRRSV-HB strain expressed in MARC-145 cells and may reflect strain-specific and host cell-dependent glycosylation patterns, which can alter electrophoretic mobility compared with the theoretical molecular weight or other strain variants [38, 39].

The knockdown efficiency of each siRNA was first confirmed by qRT-PCR (Figure 8A, B). Following PRRSV infection, viral replication was significantly increased in the negative control (NC + PRRSV) group. Notably, suppression of *CSF3* expression led to a marked reduction in viral RNA copies (Figure 8C). Similarly, knockdown of *METTL1* also significantly inhibited PRRSV replication (Figure 8D), suggesting both genes play proviral roles.

Western blot analysis further corroborated these findings at the protein level. The elevated expression of the viral GP5 protein in the NC + PRRSV group was notably attenuated upon downregulation of either *CSF3* (Figure 8E, F) or *METTL1* (Figure 8G, H), consistent with the qPCR results.

In summary, these findings demonstrate that knockdown of *CSF3* or *METTL1* expression inhibits PRRSV replication in MARC145 cells, indicating that both host genes play critical roles in supporting viral infection.

### Validation of *CSF3* and *METTL1* in porcine alveolar macrophages (PAMs)

**Figure 9:**
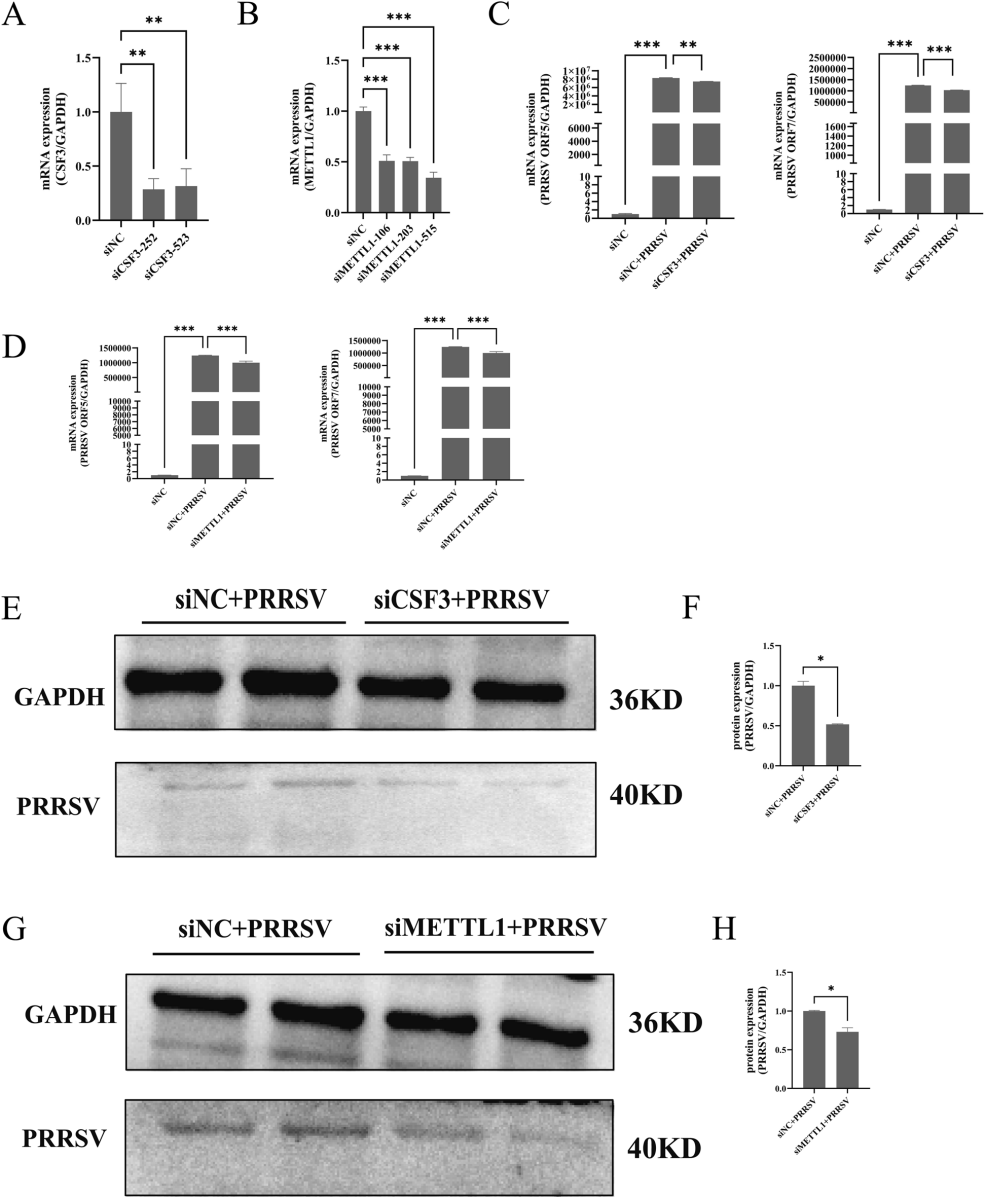
**Knockdown of** ***CSF3*** **or** ***METTL1*** **inhibits PRRSV replication in porcine alveolar macrophages (PAMs).** **A**, **B** knockdown efficiency of **A** siCSF3 and **B** siMETTL1 in PAMs, as determined by qRT-PCR. **C**, **D** PRRSV mRNA expression levels in PAMs after transfection with **C** siCSF3 or **D** siMETTL1 and infection with PRRSV (MOI = 0.1) for 36 h. **E**–**H** Western blot analysis (**E**, **G**) and corresponding grayscale quantification (**F**, **H**) of PRRSV GP5 protein levels in PAMs after knockdown of **E**, **F** *CSF3* or **G**, **H** *METTL1*. Data are presented as mean ± SD from three independent experiments. *p < 0.05, **p < 0.01, ***p < 0.001 (by Student’s *t*-test). The siRNA sequences used were siCSF3-252 and siMETTL1-523.

To confirm the biological relevance of *CSF3* and *METTL1* in the natural target cells of PRRSV, we extended our functional validation to primary porcine alveolar macrophages (PAMs). Given the potential for cell-type-specific siRNA efficacy, we employed the optimized siRNA sequences siCSF3-252 and siMETTL1-515 for PAMs, which effectively knocked down the mRNA levels of their respective target genes (Figure 9A, B). We next assessed the impact of gene knockdown on viral replication in this critical host cell. Consistent with the results from MARC145 cells, a significant increase in PRRSV RNA was detected in the control PAMs (NC + PRRSV). Crucially, suppression of either *CSF3* or *METTL1* expression led to a substantial and significant decrease in viral RNA copies (Figure 9C, D). This antiviral effect was further confirmed at the protein level by western blot analysis. The elevated expression of the viral GP5 protein in the NC + PRRSV group was markedly reduced upon knockdown of *CSF3* (Figure 9E, F) or *METTL1* (Figure 9G, H). The grayscale quantitative analysis of GP5 protein levels, normalized to β-actin, was consistent with the qPCR data.

The consistent and significant inhibition of PRRSV replication following *CSF3* or *METTL1* knockdown in primary PAMs robustly validates these genes as critical host factors supporting PRRSV infection within its physiologically relevant target cells.

## Discussion

Since its initial detection in the USA in 1987, porcine reproductive and respiratory syndrome (PRRS) has emerged as a significant threat to the global swine industry [40]. Extensive research into its pathogenic mechanisms has elucidated that PRRS virus (PRRSV) proliferation within the host relies on interactions with host cellular components. Based on these host–virus interaction mechanisms, strategies such as gene silencing, gene knockout, and the reduction or inhibition of host factor expression have been proposed to inhibit PRRSV invasion and infection [41]. Recent studies have demonstrated that targeting host cells using ATAC-seq and RNA-seq represents a novel antiviral strategy. However, there is a paucity of reports on the interactions between PRRSV and host transcription factors and genes. Moreover, no studies have yet explored the combined analysis of transcriptomics and chromatin accessibility in PRRSV-infected alveolar macrophages.

In this study, we conducted the first comprehensive analysis of chromatin accessibility and gene expression changes in PRRSV-infected porcine alveolar macrophages (PAMs) compared with controls. Our objective was to identify key host factors involved in PRRSV infection. Through integrative multi-omics analysis, we further characterized critical factors in biological processes and target genes of transcription factors. Initially, we analyzed chromatin accessibility differences between infected and control groups using ATAC-seq. Compared with controls, 6115 upregulated peaks and 2549 downregulated peaks were identified in the infected group. Among the transcription factors predicted to bind to these differential regulatory peaks, IRF1 has been associated with PRRSV infection. Research indicates that miR-296-3p targets IRF1, thereby promoting viral infection and regulating TNF-α expression in PAMs during HP-PRRSV infection. IRF1 accomplishes this by activating the IRF1 response element located on the TNF promoter [42]. To date, direct associations of Sfpi1_1, CEBPA, Fra1, NFkB-p65, RUNX1, TFE3, CREB1, and Klf1 with PRRSV have not been reported. However, these transcription factors have been implicated in other viral infections through their regulation of immune responses, cell proliferation, and viral replication-related pathways. Sfpi1_1 is linked to the mouse spleen focus-forming virus (SFFV), a retrovirus associated with Friend leukemia. The transcriptional regulation of Sfpi1_1 is driven by the long terminal repeat (LTR) of SFFV, suggesting a potential role for Sfpi1_1 in virus-related gene expression [43]. CEBPA regulates the innate immune pathway by acting on RIG-I and MDA5, thereby mediating antiviral effects [44]. Upregulation of CEBPA has been observed in infections with neural necrosis virus (NNV) and human immunodeficiency virus (HIV) [45, 46]. Fra1, a member of the Fos protein family, can regulate both innate and adaptive immunity [47]. Studies have shown that downregulating the JUN/FOS signaling cascade can inhibit PRRSV replication [48]. Viral infections typically activate the NF-κB signaling pathway. NFkB-p65 can bind to the promoter regions of virus-related genes to regulate their expression. Moreover, NFkB-p65 is involved in modulating the immune response of host cells, which is crucial for resisting viral infections. For instance, respiratory syncytial virus (RSV) captures p65, inhibits its nuclear translocation, and blocks its transcriptional activity, thereby antagonizing the host immune response and creating a favorable environment for viral infection and transmission [49]. RUNX1 is associated with inflammatory responses, and its knockdown can significantly suppress influenza A virus (IAV) infection [50], while its overexpression can inhibit herpes simplex virus type 1 (HSV-1) [51]. TFE3 plays a significant role in innate antiviral immunity, mediating GAS (Golgi apparatus stress)  responses during avian influenza virus infection [52, 53]. CREB1 is involved in the infection processes of viruses such as Ebola virus (EBOV), hepatitis B virus, and SARS-CoV-2 [54–56]. KLF1 may influence antiviral responses by regulating the expression of other genes [57]. Additionally, SpiB, a member of the E26 transformation-specific transcription factor family, coregulates B cell development and function with PU.1, and may thereby affect B cell responses to viral infections [58]. Integration of ATAC-seq and RNA-seq data revealed 1352 differentially expressed genes in the infected group compared with controls. We focused on the top 10 genes with the lowest *p*-values in the transcriptome analysis. Among these, *CCL20*, *CXCL10*, *CXCL8*, and *USP18* were associated with PRRSV infection. In PRRSV-infected porcine dendritic cells coinfected with *Streptococcus suis*, significant upregulation of proinflammatory factors such as *CCL20* was observed, indicating that heightened inflammation may contribute to disease progression during coinfection [59]. Chemokines *CXCL8* and *CXCL10*, along with their receptor CXCR3, are highly expressed in PRRSV-infected pig lungs and may represent therapeutic targets for treating PRRSV-induced lung injury [60, 61]. Porcine ubiquitin-specific protease 18 (*USP18*) acts as a host restriction factor during the innate immune response to PRRSV, with its overexpression inhibiting PRRSV replication in vitro [62].

Among the genes selected for transcriptome validation, *METTL1* and *CSF3* were identified as potentially impactful on viral infection. Current research on the direct relationship between *METTL1* and viral infection remains limited. *METTL1*, which is involved in m7G RNA modification, plays a crucial role in regulating transfer RNA (tRNA) expression, stability, and function, as well as in the regulation of disease-associated genes [63]. WDR4, a cofactor of *METTL1*, has been shown to inhibit cancer cell proliferation, migration, and invasion when silenced, while its forced expression promotes tumor progression. In the tumor immune microenvironment, m7G modification may influence tumor development by modulating immune cell infiltration and activation [64]. Future research may explore the potential role of *METTL1* in viral infections. Colony-stimulating factor 3 (*CSF3*) is also implicated in viral infections. *CSF3* and its receptor CSF3R regulate granulocyte production, neutrophil function, and hematopoietic stem cell mobilization [65]. During viral infections, CSF3 expression may be upregulated to enhance immune responses. For instance, *CSF3* is significantly upregulated in SARS-CoV-2-infected human lung A549, NHBE, and Calu-3 cells [66]. Additionally, *CSF3* is associated with immune regulatory proteins in human respiratory syncytial virus (hRSV) infection, potentially influencing virus-induced inflammation [67]. *CSF3* may affect viral pathogenesis by modulating immune cell function and inflammatory responses. Through GO enrichment and KEGG pathway analyses, we identified several canonical pathways involved in the host immune response regulated by PRRSV infection, including Kaposi’s sarcoma-associated herpesvirus infection, cytokine–cytokine receptor interaction, and chemokine signaling pathways.

The host factors identified in this study (METTL1, CSF3, IRF1, CXCL8, CXCL10) represent potential targets for developing novel antiviral strategies against PRRSV. For example, targeting METTL1 or CSF3 with small molecule inhibitors or neutralizing antibodies might suppress PRRSV replication, though this requires further validation. Furthermore, the transcription factors identified (IRF1, NFkB-p65, CEBPA) regulate key antiviral pathways and could guide vaccine adjuvant design to enhance immune responses. Additionally, genetic variants in these genes or regulatory regions could eventually support selective breeding programs for PRRSV-resistant pigs. While these applications are currently conceptual, our findings provide a foundation for future translational research aimed at improving PRRSV prevention and control.

## Conclusions

This study offers novel insights into the identification of open chromatin regions and transcription factors regulated by PRRSV infection through the integration of ATAC-seq and RNA-seq analyses. Our findings reveal substantial alterations in host gene expression induced by PRRSV and highlight several potential candidate genes associated with PRRSV infection, including *CCL20*, *CXCL10*, *CXCL8*, *USP18*, *METTL1*, and *CSF3*. Additionally, we identified key pathways such as cytokine receptor interactions and chemokine signaling pathways that are significantly impacted by PRRSV infection. These candidate genes and pathways are crucial for elucidating the mechanisms underlying PRRSV infection, and further investigation into their specific roles is warranted.

To our knowledge, this is the first study to comprehensively analyze PRRSV infection using a combined approach of ATAC-seq and RNA-seq. Our results demonstrate that several key genes in the interferon (IFN) signaling pathway, including JAK2 and STAT1, are upregulated in virus-infected hosts. This upregulation may contribute to the cellular processes associated with viral infection. Therefore, our findings provide valuable new insights into the host gene changes induced by PRRSV infection and lay a theoretical foundation for future research into the molecular mechanisms of PRRSV-host interactions.

## Data Availability

.RNA-seq and ATAC-seq data are available at ScienceDB under DOI: https://doi.org/10.57760/sciencedb.30139.
